# Prognosis and management of acute symptomatic seizures: a prospective, multicenter, observational study

**DOI:** 10.1186/s13613-023-01183-0

**Published:** 2023-09-15

**Authors:** Julia Herzig-Nichtweiß, Farid Salih, Sascha Berning, Michael P. Malter, Johann O. Pelz, Piergiorgio Lochner, Matthias Wittstock, Albrecht Günther, Angelika Alonso, Hannah Fuhrer, Silvia Schönenberger, Martina Petersen, Felix Kohle, Annekatrin Müller, Alexander Gawlitza, Waldemar Gubarev, Martin Holtkamp, Bernd J. Vorderwülbecke, IGNITE! study group

**Affiliations:** 1 Epilepsy-Center Berlin-Brandenburg, Department of Neurology with Experimental Neurology, Charité – Universitätsmedizin Berlin, corporate member of Freie Universität Berlin and Humboldt-Universität zu Berlin, Berlin, Charitéplatz 1, 10117 Germany; 2 Department of Neurology, Klinikum Osnabrück, Osnabrück, Germany; 3 Department of Neurology, Faculty of Medicine, University of Cologne and University Hospital of Cologne, Cologne, Germany; 4 Department and Policlinic of Neurology, Leipzig University Medicine, Leipzig, Germany; 5 Department of Neurology, Medical Faculty, Saarland University Medical Center, Homburg a. d. Saar, Germany; 6 Department and Policlinic of Neurology, Rostock University Medical Center, Rostock, Germany; 7 Department of Neurology, University Hospital Jena, Jena, Germany; 8 Department of Neurology, Medical Faculty Mannheim, Ruprecht Karl University of Heidelberg, Mannheim, Germany; 9 Department of Neurology, University Hospital Freiburg, Freiburg, Germany; 10 Department of Neurology, Medical Faculty Heidelberg, Ruprecht Karl University of Heidelberg, Heidelberg, Germany

**Keywords:** Acute symptomatic seizure, Antiseizure medication, Cerebrovascular accident, CNS infection, Ischemic stroke, Mortality, Secondary seizure prophylaxis, Seizure risk, Structural brain damage, Unprovoked seizure

## Abstract

**Background:**

Acute symptomatic epileptic seizures are frequently seen in neurocritical care. To prevent subsequent unprovoked seizures, long-term treatments with antiseizure medications are often initiated although supporting evidence is lacking. This study aimed at prospectively assessing the risk of unprovoked seizure relapse with respect to the use of antiseizure medications. It was hypothesized that after a first acute symptomatic seizure of structural etiology, the cumulative 12-month risk of unprovoked seizure relapse is ≤ 25%.

**Methods:**

Inclusion criteria were age ≥ 18 and acute symptomatic first-ever epileptic seizure; patients with status epilepticus were excluded. Using telephone and mail interviews, participants were followed for 12 months after the acute symptomatic first seizure. Primary endpoint was the occurrence and timing of a first unprovoked seizure relapse. In addition, neuro-intensivists in Germany were interviewed about their antiseizure treatment strategies through an anonymous online survey.

**Results:**

Eleven of 122 participants with structural etiology had an unprovoked seizure relapse, resulting in a cumulative 12-month risk of 10.7% (95%CI, 4.7%–16.7%). None of 19 participants with a non-structural etiology had a subsequent unprovoked seizure. Compared to structural etiology alone, combined infectious and structural etiology was independently associated with unprovoked seizure relapse (OR 11.1; 95%CI, 1.8–69.7). Median duration of antiseizure treatment was 3.4 months (IQR 0–9.3). Seven out of 11 participants had their unprovoked seizure relapse while taking antiseizure medication; longer treatment durations were not associated with decreased risk of unprovoked seizure relapse. Following the non-representative online survey, most neuro-intensivists consider 3 months or less of antiseizure medication to be adequate.

**Conclusions:**

Even in case of structural etiology, acute symptomatic seizures bear a low risk of subsequent unprovoked seizures. There is still no evidence favoring long-term treatments with antiseizure medications. Hence, individual constellations with an increased risk of unprovoked seizure relapse should be identified, such as central nervous system infections causing structural brain damage. However, in the absence of high-risk features, antiseizure medications should be discontinued early to avoid overtreatment.

**Supplementary Information:**

The online version contains supplementary material available at 10.1186/s13613-023-01183-0.

## Introduction

Following the International League Against Epilepsy (ILAE), ‘an acute symptomatic seizure is defined as a clinical seizure occurring at the time of a systemic insult or in close temporal association with a documented brain insult’ [1]. Approximately 2–3% of patients in intensive care and even 8–11% of those in neurocritical care have acute symptomatic seizures [2]. Overall, in cerebrovascular disease, incidence ranges from 1.3% in ischemic stroke [3] to 4% in intracerebral or subarachnoid hemorrhage [4] up to 34% in cerebral venous thrombosis [5]. Acute symptomatic seizures may indicate an increased risk of subsequent unprovoked seizures [6–9] but nevertheless, they must be distinguished from epilepsy which is defined by recurrent unprovoked seizures [10]. A hallmark study on first seizures after stroke, traumatic brain injury or central nervous system (CNS) infections found that the cumulative 10-year risk of unprovoked seizure relapse after a first *acute symptomatic* seizure was 19% (95%CI: 14–25%), compared to 65% (55–74%) after a first *unprovoked* seizure [11]. Based on this discrepancy, current clinical guidelines advise against initiating long-term antiseizure treatments after acute symptomatic seizures [12–14], while a 10-year seizure relapse risk of > 60% defines epilepsy and justifies long-term medical treatment [10].

However, patients included in the abovementioned study had their index seizures between 1955 and 1984, when modern clinical guidelines were not available. Data collection was retrospective, and data on antiseizure medications as potential confounders of seizure outcome were not on hand [11]. This calls for new, prospective studies to estimate the seizure relapse risk of patients treated according to current guidelines. Admittedly, these guidelines are rarely followed: In intensive care, the majority of antiseizure treatments started due to acute symptomatic seizures are continued beyond discharge from the intensive care unit [2]. Once a patient is discharged from the hospital with ongoing medication, it is likely that the treatment is continued for many months or even years [15, 16].

To confirm that acute symptomatic seizures bear a low risk of subsequent unprovoked seizures even in presence of a structural brain pathology, the multicenter ‘Register on the PROgnosis of acute symptomatic SEizures’ (PROSE register) was initiated. PROSE aimed at following up on patients with first acute symptomatic seizures, accounting for the use of antiseizure medications. Based on previous evidence [11], it was hypothesized that after a first acute symptomatic seizure of structural etiology, the 12-month risk for unprovoked seizure relapse is not higher than 25%, even if patients were not treated with antiseizure medications, or for a short period only. Secondarily, it was hypothesized that acute symptomatic seizures due to structural brain lesions have a higher risk of subsequent unprovoked seizures than acute symptomatic seizures with a non-structural etiology. In addition, an online survey among neurologists, neurosurgeons, and intensivists in Germany was conducted to explore real-life antiseizure treatment strategies following acute symptomatic seizures.

## Methods

### Study design and sample size estimation

The PROSE register is an investigator-initiated, prospective, open, single-arm, observational study with ongoing follow-up for up to 3 years [17]. It is conducted as a multicenter study within the ‘Initiative of German Neuro-Intensive Trial Engagement’ (IGNITE!) study network of the German Neurocritical Care Society (DGNI). Sample size was estimated to confirm the primary study hypothesis, adjusting for age and competing events [18]. Assuming a censoring rate of 20% (death or loss to follow-up) and a true event rate of 15% (unprovoked seizure relapse) with a 95%CI below 25% (8.3%–23.1%), 115 patients with acute symptomatic first-ever seizures of structural etiology needed to be recruited. As the secondary hypothesis was exploratory, no minimum number of participants with non-structural etiologies was specified. To increase the sample size, participants from a monocenter pilot phase at Charité – Universitätsmedizin Berlin were included into the study cohort. Study protocols during the mono- and multicenter phases were identical, except that screening failures were documented during the multicenter recruitment phase only.

### Inclusion and exclusion criteria

Inclusion criteria were as follows: Age ≥ 18 years and evidence of an acute symptomatic first-ever seizure following the ILAE criteria [1] within 14 days prior to inclusion. In addition to constellations covered by the ILAE definition [1], seizures during posterior reversible encephalopathy syndrome (PRES), cerebral edema due to reperfusion syndrome, and sepsis were also considered acute symptomatic. Although febrile seizures typically occur in children, fever of > 40.0 °C (> 104.0 °F) was accepted as a further potential cause of acute symptomatic seizures in adults. Patients with a history of previous seizures were not eligible. Status epilepticus as defined by the ILAE [19] was an exclusion criterion because of the increased risk of subsequent unprovoked seizures [9, 20].

### Data collection

Recruitment efforts were focused on, but not restricted to, intensive and intermediate care units. At the time point of inclusion into the study, details on the seizure and the underlying pathology were documented. Referring to the ILAE classification of the epilepsies [21], etiology was considered structural if the pathology was traumatic or cerebrovascular, and/or if an affection of brain tissue was evident from neuroimaging (e.g., in meningoencephalitis). Presence of sepsis [22], the current modified Rankin Score (mRS) as a measure of functional outcome [23], and the Simplified Acute Physiology Score II (SAPS II) as an estimate of overall disease severity [24] were documented. Participants were re-evaluated at discharge from the intensive care unit (if applicable) and at discharge from the acute care hospital. Three, 6, and 12 months after the acute symptomatic seizure, participants or their next of kin were interviewed via telephone; those not reached by telephone were contacted by mail.

The study’s primary outcome parameter was occurrence and time point of a first unprovoked seizure relapse within 12 months after the acute symptomatic seizure. To increase sensitivity, i.e., to avoid missing a potential seizure, participants were asked three structured questions modified from reference [25] (Additional file 1: Table S1). To ensure high specificity, i.e., to rule out seizure mimics, once a screening question was answered in the affirmative, the participant was re-evaluated by a neurologist. Secondary outcome parameters were occurrence and time point of additional acute symptomatic seizures, use of sedatives or antiseizure medications, seizure-related rehospitalization, and current overall functional outcome according to mRS. Pseudonymized participant data were collected and managed using Research Electronic Data Capture (REDCap) tools hosted at Charité – Universitätsmedizin Berlin [26, 27].

### Study registration

Prior to the multicenter recruitment phase, the study was prospectively registered in the German Clinical Trials Register (ID: DRKS00017811).

### Online survey

As an addendum to the main study, a non-representative, anonymous online survey on antiseizure treatment in clinical practice was conducted between 21st February and 8th April 2022. Invitations to participate were disseminated through the e-mail newsletter of the DGNI and within centers participating in the PROSE register. Participants had to confirm that they worked as physicians. They were asked for their field of specialty, and whether or not they knew the PROSE register. Three fictitious cases of a first acute symptomatic seizure due to PRES/eclampsia, ischemic stroke, and herpes simplex virus type-1 encephalitis were given, plus one fictitious case of a first unprovoked seizure following traumatic brain injury. Participants were asked for how long they would recommend antiseizure treatment, with five options ranging from ‘not at all’ to ‘permanently’. In addition, they were asked in which case(s) they would have an electroencephalogram (EEG) performed acutely and/or during follow-up to guide their treatment decision (Additional file 1: Table S2). The survey was conducted using a REDCap tool hosted at Charité – Universitätsmedizin Berlin [26, 27].

### Statistical analyses

Data were analyzed using SPSS 29 (IBM, Armonk, US-NY). Data are given as percent or as median and interquartile range (IQR). The cumulative risk of unprovoked seizure relapse within 12 months was estimated using the Kaplan–Meier method. The 95%CI of the Kaplan–Meier estimator was calculated as (estimator ± 1.96* standard error); factors were compared using the log-rank test. For bivariate group comparisons, nominal data in 2 × 2 tables were subjected to Fisher’s exact test, other nominal data were compared using *χ*^2^ test. Ordinal or continuous data from independent samples were subjected to the Mann–Whitney *U* test; ordinal data from dependent samples were subjected to the Wilcoxon test. All tests were two-sided; *p* < 0.05 was considered statistically significant. Since all subgroup analyses were exploratory, no post-hoc correction was performed.

Among PROSE cases with structural etiology, variables with *p* < 0.1 were subjected to a binary logistic regression (method: inclusion; iteration 20; constant included) to identify parameters independently associated with treatment with antiseizure medications for > 100 days and with 12-month seizure relapse, respectively. To estimate the effect of prolonged treatment on unprovoked seizure relapse risk despite lack of randomization, following binary logistic regression, a generalized estimating equation was generated with each case weighted by their inverse probability of being treated for > 100 days (propensity score) [28].

## Results

### Study cohort

**Fig. 1:**
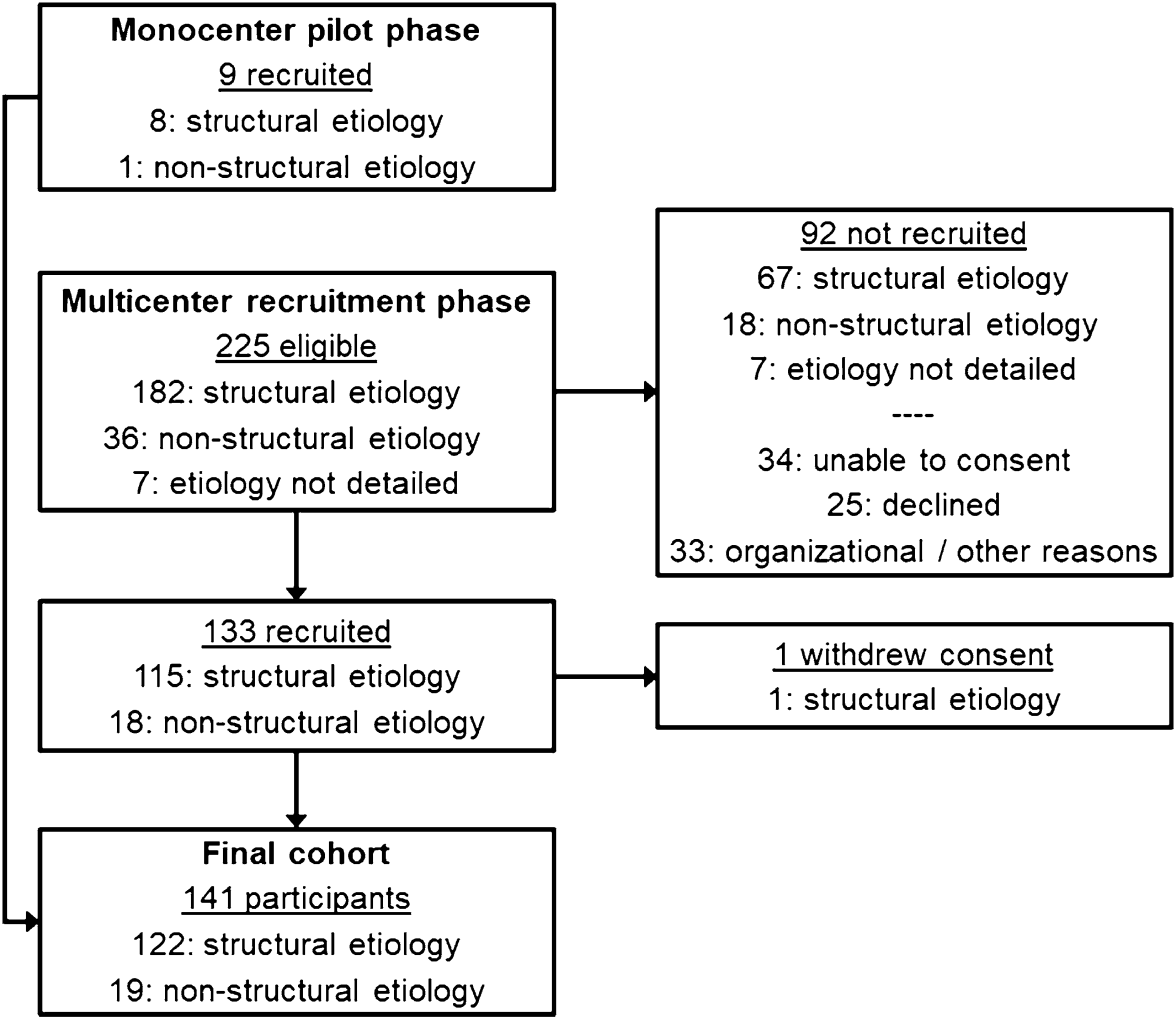
Flowchart of participant recruitment for the PROSE register. The multicenter recruitment phase was preceded by a monocenter pilot phase at Charité – Universitätsmedizin Berlin. During that monocenter pilot phase, eligible but not successfully recruited patients (‘screening failures’) were not documented. Otherwise, the study protocol was identical

Throughout the monocenter pilot phase from May to August 2019, nine patients were recruited at Charité – Universitätsmedizin Berlin. During the multicenter recruitment phase from September 2019 to July 2021, 133 of 225 eligible patients (59%; Fig. 1) were recruited in 10 centers of the IGNITE! network. Recruited patients were significantly younger (median 62 years; IQR 52–75) than those not recruited (median 69 years; IQR 59–80; *p* = 0.009; Additional file 1: Table S3). One legal guardian later withdrew approval and requested deletion of the participant’s data. Eventually, the study cohort consists of 141 subjects with first acute symptomatic seizures, 122 of these (87%) with structural etiology.

**Fig. 2:**
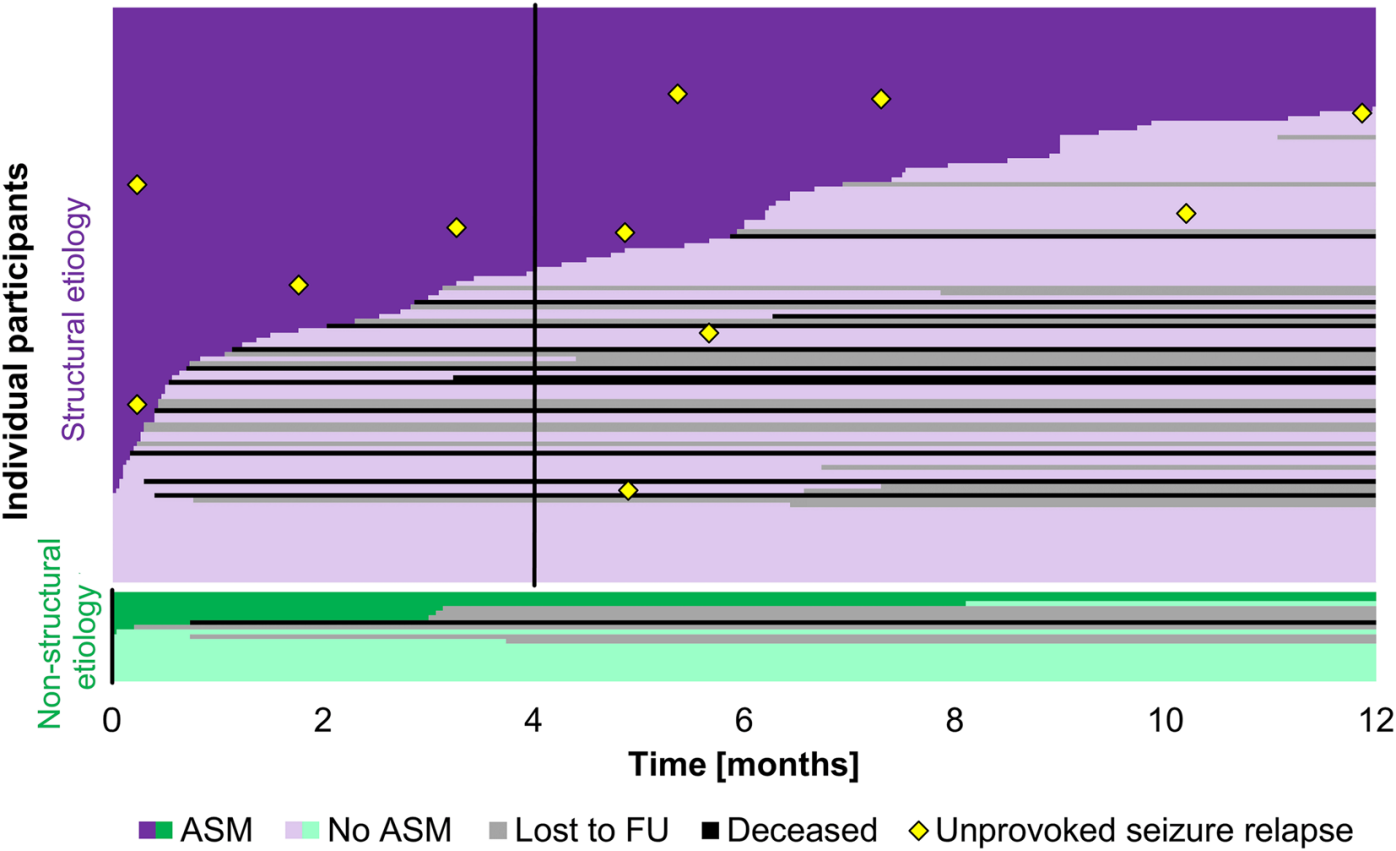
Durations of antiseizure treatment following acute symptomatic seizures. Durations of initial treatment with antiseizure medication (ASM; dark color) followed by time without antiseizure medication (bright color) in individual participants with acute symptomatic seizure of structural etiology (purple, *n* = 122) vs. non-structural etiologies (green, *n* = 19). Grey and black, ASM intake could not be determined because of loss to follow-up (FU) or death. Yellow rhombus, unprovoked seizure relapse. Black vertical lines, median durations of initial ASM treatment were 4.0 months in cases with structural etiology (*n* = 106) and 0 months in cases with non-structural etiology (*n* = 14)

Details on the study cohort and the acute symptomatic seizures are given in Table 1, while underlying pathologies are detailed in Table 2. Among structural pathologies, cerebrovascular accidents formed the most prevalent group (*n* = 90; 74%), followed by infections with structural affection of brain tissue visible on neuroimaging (*n* = 14; 11%). Among patients with non-structural pathologies, 15 (79%) underwent cerebral magnetic resonance imaging (MRI); three patients with alcohol withdrawal syndrome and one with severe hypocalcemia had a computed tomography scan. Fifty-three participants (38%) had more than one seizure during the acute phase. A median of 2 days (IQR 1–5) after the first seizure, 121 participants (86% Cf table 1) had an electroencephalogram (EEG) which showed epileptiform activity in 13 (11%) of them. Within 12 months of the acute symptomatic seizure, 13 PROSE participants died (9%), and another 22 were lost to follow-up (16%; Fig. 2). The median follow-up time was 12 months (IQR 6.9–12). From inclusion to 12 months, the median mRS dropped from 3 (IQR: 1–5) to 0 (0–3; *p* < 0.001; Additional file 1: Figure S1).

**Table 1:** Overview on participants and acute symptomatic seizures

		Structural etiology, *n* = 122	Non-structural etiology, *n* = 19	*p*, uncorrected
Sex	Female	54 (44%)	5 (26%)	0.21
	Male	68 (56%)	14 (74%)	
Age [years]		63 (54–76)	56 (46–75)	0.14
Inpatient treatment	ICU	64 (52%)	4 (21%)	< 0.001
	IMC	16 (13%)	4 (21%)	
	Stroke unit/telemetry unit	36 (30%)	3 (16%)	
	General ward	6 (5%)	8 (42%)	
Mechanical ventilation	Ventilation	43 (35%)	2 (11%)	0.03
	No ventilation	79 (65%)	17 (89%)	
Sepsis	Sepsis	6 (5%)	3 (16%)	0.10
	No sepsis	116 (95%)	16 (84%)	
SAPS II		26 (19–35); n = 39*	28 (15–36); n = 6*	0.64
Initial mRS		3 (1–5)	1 (0–3)	0.19
Acute symptomatic seizure as initial symptom of underlying pathology	Initial symptom	64 (52%)	14 (74%)	0.14
	Not initial symptom	58 (48%)	5 (26%)	
Delay between manifestation of underlying pathology and acute symptomatic seizure	< 24 h	85 (70%)	17 (90%)	0.10
	> 24 h	37 (30%)	2 (10%)	
Acute symptomatic seizure type	Tonic–clonic	84 (69%)	16 (84%)	0.28
	Other than tonic–clonic	38 (31%)	3 (16%)	
Single vs. multiple acute symptomatic seizures	Single seizure	74 (61%)	14 (74%)	0.35
	Multiple, within 24 h after first seizure	26 (21%)	4 (21%)	
	Multiple, beyond 24 h after first seizure	22 (18%)	1 (5%)	
Inpatient EEG	Epileptiform activity	12 (10%)	1 (5%)	0.69
	No epileptiform activity	92 (75%)	16 (84%)	
	EEG not performed	18 (15%)	2 (11%)	

Data are given as *n* (column %) or median (interquartile range). *p* values < 0.05 are underlined. ICU, intensive care unit. IMC, intermediate care unit. SAPS II, simplified acute physiology score II. mRS, modified Rankin score. MRI, magnetic resonance imaging. CT, computed tomography. EEG, electroencephalogram. * Otherwise unknown

**Table 2:** Pathologies underlying acute symptomatic seizures

Structural etiology			Non-structural etiology	
Pathology	Cases	Unprovoked seizure relapse	Pathology	Cases
*All*	*122 (100%)*	*11 (100%)*	*All*	*19 (100%)*
Ischemic stroke	37 (30%)	2 (18%)	Alcohol withdrawal syndrome	7 (37%)
Intracerebral hemorrhage	23 (19%)	2 (18%)	Infectious meningitis	5 (26%)
Cerebral venous thrombosis	14 (11%)	2 (18%)	Electrolyte imbalance ^*^	2 (11%)
Subarachnoid hemorrhage	13 (11%)	0	Sepsis	2 (11%)
PRES/eclampsia	11 (9%)	0	Autoimmune encephalitis	1 (5%)
Viral encephalitis	7 (6%)	2 (18%)	Fever > 40.0 °C	1 (5%)
Bacterial meningoencephalitis	5 (4%)	1 (9%)	Viral encephalitis	1 (5%)
Autoimmune encephalitis	3 (2%)	0		
Cerebral abscess	2 (2%)	1 (9%)		
Hyperperfusion syndrome	2 (2%)	0		
Subdural hematoma	2 (2%)	0		
Hypoxic–ischemic encephalopathy	1 (1%)	1 (9%)		
Transient ischemic attack	1 (1%)	0		
Traumatic brain injury	1 (1%)	0		

Data are given as *n* (column %). No participant with a non-structural etiology of their acute symptomatic seizure had an unprovoked seizure relapse. PRES, posterior reversible encephalopathy syndrome

^*^Hyponatremia, hypocalcemia

### Antiseizure medications

During inpatient treatment in the acute care hospital, 121 participants (86%) received seizure-suppressing compounds. Seventy-eight were treated with sedatives, most frequently propofol (*n* = 30; 38%), lorazepam, and midazolam (*n* = 29 each; 37% each), while 108 received classical antiseizure medications, particularly levetiracetam (*n* = 106; 98%), followed by lacosamide (*n* = 14; 13%). Twenty-three participants (16%) received two or more antiseizure compounds simultaneously or successively; one was on continuous treatment with pregabalin due to anxiety disorder.

Among the 120 participants who were successfully interviewed 3 months after the acute symptomatic seizure, 67 (56%) had continued taking their antiseizure medication beyond discharge from the acute care hospital. As far as verifiable, median duration of the initial antiseizure treatment (until discontinuation or first unprovoked seizure) was 4.0 months (IQR 0.2–9.0; *n* = 106) in cases with structural etiology, and 0 months (IQR 0–2.0/0 weeks, IQR 0–8.8; *n* = 14) in cases with non-structural etiology (Fig. 2). As far as is known, 58 participants took antiseizure medications for less than 100 days while another 59 took them for more than 100 days despite freedom from unprovoked seizures. Following multivariable analysis, treatment durations of > 100 days were independently associated with administration of classical antiseizure medications in the acute care hospital, and with an inpatient EEG irrespective of epileptiform discharges (Additional file 1: Table S5).

### Unprovoked seizure relapse

**Fig. 3:**
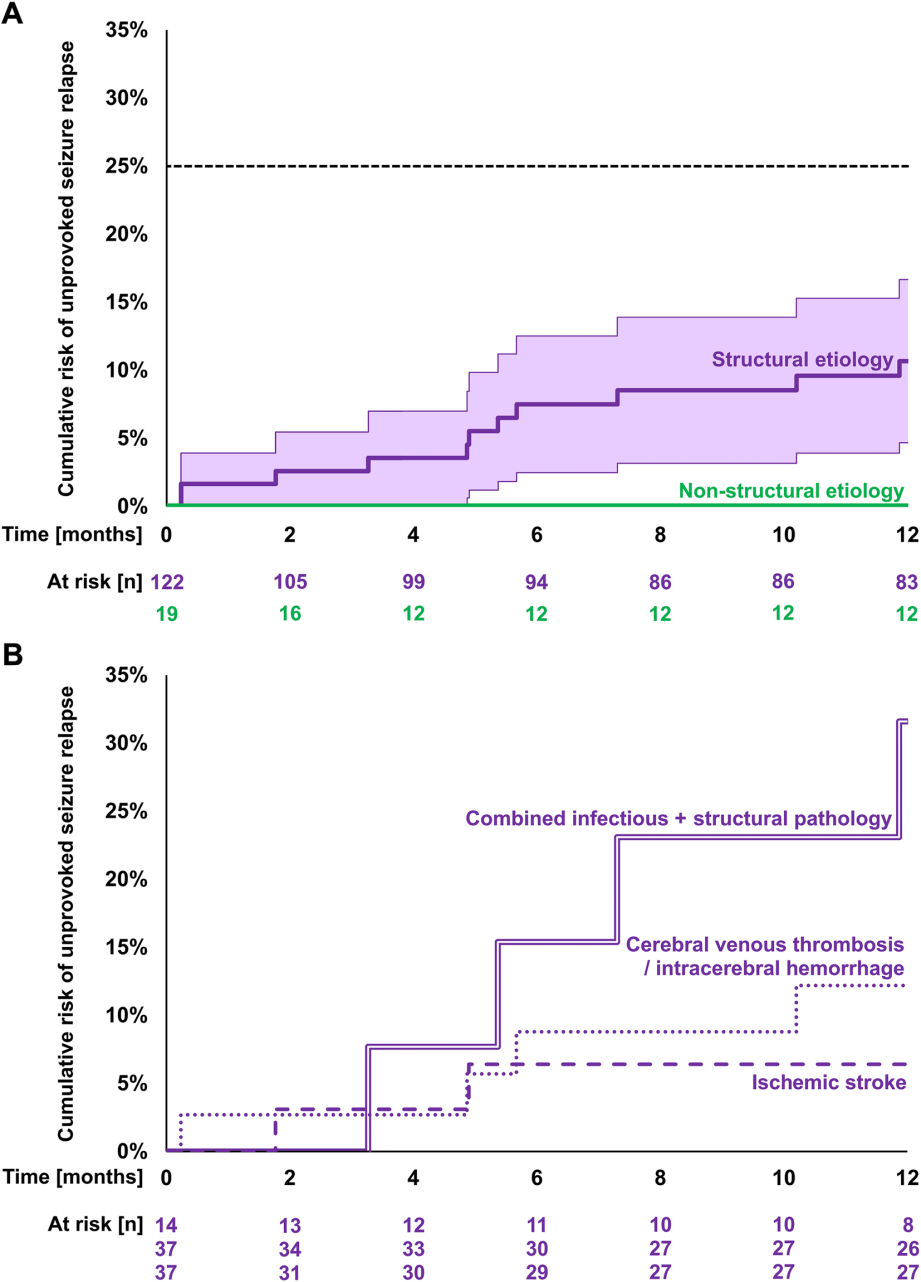
Cumulative risk of unprovoked seizure relapse – reverse Kaplan–Meier plots. **A** cumulative risk of unprovoked seizure relapse within 12 months of acute symptomatic first seizure. Purple, structural etiology with 95%CI; green, non-structural etiology. Uncorrected log-rank test, *p* = 0.22. Dashed horizontal line, a cumulative risk of ≤ 25% was initially hypothesized. **B** cumulative risk of unprovoked seizure relapse depending on the underlying structural pathology. Double line, combined infectious and structural pathology (e.g., cerebral abscess or meningoencephalitis with MR-visible affection of brain tissue; *n *= 14); dotted line, cerebral venous thrombosis and/or intracerebral hemorrhage (*n* = 37); dashed line, ischemic stroke (*n* = 37). Uncorrected log-rank test, *p* = 0.097

After a median time of 4.9 months (range 7–356 days), 11 participants with structural etiology of their acute symptomatic seizure had an unprovoked seizure relapse (Table 2; Additional file 1: Table S4); eight of them were re-hospitalized. The resulting cumulative 12-month risk of unprovoked seizure relapse was 10.7% (95%CI: 4.7%-16.7%; Fig. 3A). For comparison, no participant with a non-structural etiology had an unprovoked seizure relapse (*p* = 0.22). The cumulative 12-month risk of unprovoked seizure relapse was 6.4% (0–14.8%) in ischemic stroke, 12.2% (1.0%–23.3%) in intracerebral hemorrhage and cerebral venous thrombosis taken together, and 31.6% (5.9%–57.3%) in CNS infections with structural affection of brain tissue (*p* = 0.097; Fig. 3B).

Further details on variables associated with unprovoked seizure relapse related to structural pathologies are given in Table 3; for all etiologies, see Additional file 1: Table S6. Following multivariable analysis, combined infectious and structural pathology was independently associated with unprovoked seizure relapse (OR 11.1; 95%CI 1.8–69.7; *p* = 0.010) as compared to structural pathology alone. One formally unprovoked seizure relapse occurred while the subject had coronavirus disease 2019; in a further case, the seizure relapse led to the detection of a malign brain tumor.

**Table 3:** Variables associated with unprovoked seizure relapse after a first acute symptomatic seizure of structural etiology

		No unprovoked seizure relapse, *n* = 111	Unprovoked seizure relapse, *n* = 11	*p*, bivariate, uncorrected	Odds ratio, multivariable*	*p*, multivariable*
Sex	Female	52 (47%)	2 (18%)	0.11		
	Male	59 (53%)	9 (82%)			
Age [years]		64 (54–78)	58 (53–64)	0.20		
Inpatient treatment	ICU	59 (53%)	5 (46%)	0.83		
	IMC	15 (14%)	1 (9%)			
	Stroke unit/telemetry unit	32 (29%)	4 (36%)			
	General ward	5 (5%)	1 (9%)			
Mechanical ventilation	Ventilation	39 (35%)	4 (36%)	1.0		
	No ventilation	72 (65%)	7 (64%)			
Sepsis	Sepsis	5 (5%)	1 (9%)	0.44		
	No sepsis	106 (95%)	10 (91%)			
Initial mRS		3 (1–5)	3 (1–5)	–		
Etiology of acute symptomatic seizure	Structural only	101 (91%)	7 (64%)	0.023	1	0.010
	Structural + infectious	10 (9%)	4 (36%)		11.1 (1.8–69.7)	
Acute symptomatic seizure as initial symptom of underlying pathology	Initial symptom	61 (55%)	3 (27%)	0.11		
	Not initial symptom	50 (45%)	8 (73%)			
Delay between manifestation of underlying pathology and seizure	< 24 h	81 (73%)	4 (36%)	0.018	1	0.12
	> 24 h	30 (27%)	7 (64%)		3.0 (0.8–12.2)	
Acute symptomatic seizure type	Tonic–clonic	79 (71%)	5 (45%)	0.095	1	0.068
	Other than tonic–clonic	32 (29%)	6 (55%)		4.9 (0.9–26.9)	
Single vs. multiple acute symptomatic seizures	Single seizure	66 (59%)	8 (72%)	0.57		
	Multiple, within 24 h after first seizure	25 (23%)	1 (9%)			
	Multiple, beyond 24 h after first seizure	20 (18%)	2 (18%)			
Inpatient medication	No medication	14 (13%)	0	0.28		
	Sedatives only	9 (8%)	0			
	Classical antiseizure medication only	35 (31%)	6 (55%)			
	Sedatives + classical antiseizure medication	53 (48%)	5 (45%)			
Inpatient EEG	Epileptiform activity	10 (9%)	2 (18%)	0.25		
	No epileptiform activity	83 (75%)	9 (82%)			
	EEG not performed	18 (16%)	0			
Time to discontinuation of ASM [months]		4.0 (0.1–9.4); n = 95**	3.9 (0.4–9.9); n = 4***	0.98		

Data are given as n (column percent) or median (interquartile range). *p* values < 0.05 are underlined. mRS, modified Rankin score; ASM, antiseizure medication

*Binary logistic regression; 122 cases included; Nagelkerke’s *R*^2^ = 0.24

**Otherwise, unknown

***Otherwise, unprovoked seizure relapse at 3.3 months (0.2–5.4) while still on initial treatment

Seven out of 11 participants had their unprovoked seizure relapse after 3.3 months median (IQR 0.2–5.4) while still taking antiseizure medication (Fig. 2); in one of these, the dose was currently being reduced. The remaining four participants had discontinued their medication after 3.9 months (0.4–9.9), and the unprovoked seizure occurred at 7.9 months (5.5–10.5). Following inverse propensity score weighting, treatment with antiseizure medications for > 100 days was not associated with reduced risk of later unprovoked seizures (OR 1.2; 95%CI 0.2–6.5; *p* = 0.86).

### Online survey

**Fig. 4:**
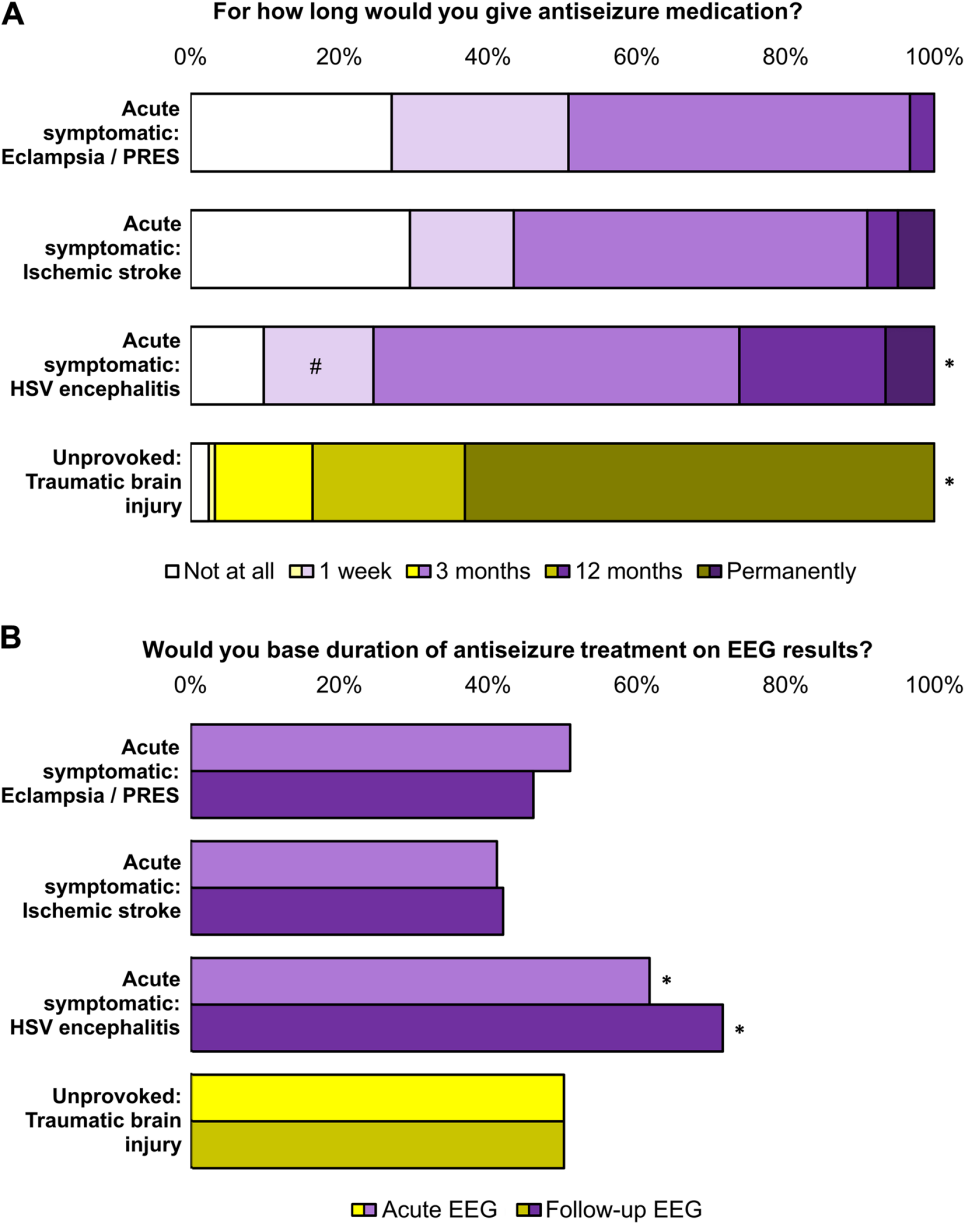
Results of the online survey on antiseizure treatment strategies. In an anonymous online survey, 122 participating physicians working in neurology, neurosurgery, and/or intensive care were given three fictitious cases of a first acute symptomatic seizure (purple) due to eclampsia/posterior reversible encephalopathy syndrome (PRES), ischemic stroke, and herpes simplex virus (HSV) type-1 encephalitis, plus one fictitious case of a first unprovoked seizure (yellow) following traumatic brain injury. **A** Recommendations for durations of treatment with antiseizure medication. **B** Demand for acute (above) and/or follow-up EEG (below) to guide decisions on treatment duration. ^#^ In case of HSV encephalitis, the ‘1 week’ option was replaced by ‘as long as anti-infective treatment is given’. * Uncorrected Wilcoxon test, *p* < 0.05 in pairwise comparisons to all other cases

One-hundred sixty participants started answering the online survey, and 122 (76%) completed it. Among these, 48 (39%) were familiar with the PROSE register. Ninety-six (79%) worked in the field of neurology, 12 (10%) in neurosurgery, and 38 (31%) in intensive care (multiple answers were allowed). Depending on the underlying pathology of acute symptomatic seizures, 74–97% recommended antiseizure treatment for 3 months or less (Fig. 4A). The longest treatment durations were recommended in case of herpes simplex virus encephalitis (*p* < 0.05). After a first unprovoked seizure following traumatic brain injury, 63% recommended permanent antiseizure treatment (*p* < 0.05). Physicians aware of the PROSE register tended to treat acute symptomatic seizures shorter and unprovoked first seizures longer than those to whom the study was unknown, but differences were not statistically significant (*p* > 0.05; Additional file 1: Figure S2). In the acute phase, 41–61% would have an EEG performed to guide their therapy, and 42–71% recommended a follow-up EEG. The highest demand for both acute and follow-up EEG was seen in herpes simplex virus encephalitis (*p* < 0.05; Fig. 4B).

## Discussion

In the PROSE cohort, the 12-month risk of unprovoked seizure relapse following acute symptomatic seizures of structural etiology was 10.7% (4.7%–16.7%) and thus significantly below 25%, as hypothesized. In cases with non-structural etiology, the risk was even zero, but the study was not powered to detect statistically significant differences between structural and non-structural etiologies. CNS infections with MRI-visible affection of brain tissue were associated with an increased risk of subsequent unprovoked seizures. Antiseizure medications were given for longer periods of time than recommended by current guidelines, but treatment durations did not affect the risk of unprovoked seizure relapse.

### Mortality and seizure outcome

In comparison to unprovoked seizures, acute symptomatic seizures are known to be associated with a high short-term mortality due to the underlying pathology but with a low risk of subsequent unprovoked seizures. In a retrospective cohort of 262 patients with acute symptomatic first seizures, 21% died within the first 30 days [11]. Among PROSE participants, 9% died within 12 months, while another 16% were lost to follow-up, and it is unclear how many of those deceased as well. The cumulative 12-month risk of unprovoked seizure relapse was 10.7%. For comparison, in the former study, it was approximately 13%. In that study, at 10 years, the cumulative risk of unprovoked seizure relapse was higher in stroke (33%) than in CNS infections (17%) and traumatic brain injury (13%). However, stroke was not differentiated into ischemic or hemorrhagic, and CNS infections were not distinguished into those with and those without structural affection of brain tissue [11]. In the current study, these differentiations appeared highly relevant (Fig. 3).

In the PROSE cohort, ischemic stroke was the most prevalent structural pathology and was associated with a cumulative 12-month unprovoked seizure relapse risk of 6.4%. A recent retrospective study including 182 patients with acute symptomatic seizures in ischemic stroke found a 12-month risk of approximately 9% [9]. Two other studies reported 12-month risks of 16% [29] and 23% [6], but these did not differentiate between acute symptomatic short seizures and status epilepticus. Acute symptomatic status epilepticus, however, increases the risk of unprovoked subsequent seizures three- to fourfold [9, 20].

In two retrospective studies on cerebral venous thrombosis and intracerebral hemorrhage with median follow-up times of 2 and 2.7 years, rates of unprovoked seizure relapse were 22% and 27% among patients with acute symptomatic seizures [7, 30]. In the current study, after 12 months, rates were 14% in cerebral venous thrombosis and 9% in intracerebral hemorrhage (Table 2). Regarding CNS infections, the 20-year risk of unprovoked seizure relapse was found to be 13% in bacterial meningitis and 22% in viral encephalitis [8], while in another study, 41% of patients with acute symptomatic seizures due to CNS infection developed pharmacoresistant focal epilepsy, with status epilepticus as an independent risk factor [31]. In the PROSE cohort, CNS infections causing structural brain damage brought along a high risk of subsequent unprovoked seizures not only in comparison to CNS infection without structural brain damage, but also in comparison to structural pathologies without infection. In acute brain damage, inflammation is a known risk factor for epileptogenesis [32]. Therefore, special caution should be given to cases with combined infectious and structural etiology in the future. On the other hand, seizures caused by non-structural pathologies have no apparent risk of subsequent unprovoked seizures, and the term ‘acute symptomatic’ has even been questioned for these cases [33].

Apart from one abovementioned work related to ischemic stroke [29], other prospective studies assessing the prognosis of acute symptomatic seizures are rare. One study followed 105 patients with symptomatic first seizures; however, CNS tumors, mitochondrial disease and status epilepticus were included. The risk of unprovoked seizure relapse was 5% within one year and 12% within two years [34]. In a recent study on 305 infants with neonatal acute symptomatic seizures of structural etiology, 13% developed epilepsy, irrespective of whether antiseizure medications were withdrawn early or not [35].

### Clinical management

It seems intuitive that the use of antiseizure medications influences the risk of seizure occurrence. Nevertheless, data on the relation between prognosis of acute symptomatic seizures and antiseizure medications in adults are sparse. Among PROSE participants, 86% received sedatives or classical antiseizure medications in the acute care hospital, 57% continued their antiseizure medication beyond discharge, and 51% were treated for more than 3 months. In the subgroup of participants with ischemic stroke, 73% received an initial antiseizure treatment; this is comparable to 52–86% in three previous studies [6, 9, 29].

Short-term medical treatments of acute symptomatic seizures appear reasonable because, as in the current cohort, 30–40% of patients have multiple seizures during the acute phase of the underlying condition [34]. In a randomized controlled trial, phenytoin as a primary prophylaxis prevented acute symptomatic seizures following traumatic brain injury, but not unprovoked subsequent seizures [36]. There is currently no evidence that generally favors long-term antiseizure treatment after acute symptomatic seizures. Consequently, most clinical guidelines recommend no antiseizure treatment or short-term treatment during the acute phase only [12–14], while others do not provide any recommendation [37, 38].

According to the non-representative online survey described above, neurologists, neurosurgeons and intensivists in Germany deem a treatment duration of 3 months most appropriate, depending on the underlying pathology (Fig. 4A). Three months is by far longer than the time window defining acute symptomatic seizures in structural pathologies, which is usually 7 days [1]. In the PROSE cohort, most treatment durations were even longer than 3 months (Fig. 2), underlining the overall tendency towards medical overtreatment of acute symptomatic seizures. In acute care settings, acute symptomatic seizures are not sufficiently distinguished from unprovoked seizures [2], and once a patient is discharged with an antiseizure treatment, physicians in outpatient care are reluctant to discontinue the medication [15, 16]. Indeed, uncritical use should be avoided because antiseizure medications can cause a plethora of adverse effects [39].

In the PROSE cohort, epileptiform EEG activity in the acute phase was not found to be associated with unprovoked seizure relapse. However, following the online survey, about half of physicians consider EEG results helpful for their treatment decisions both in acute symptomatic and unprovoked seizures (Fig. 4B). While after a first unprovoked seizure, epileptiform EEG activity is known to indicate a high risk of subsequent seizures [40], the prognostic value of EEG abnormalities in acute symptomatic seizures is less clear. In retrospective cohorts, epileptiform activity in acute EEG was found to be associated with further acute symptomatic seizures [34], whereas epileptiform activity in follow-up EEG was found to be associated with unprovoked seizure relapse [41]. In general, evidence for the prognostic value of EEG abnormalities in acute symptomatic seizures is very low.

Unprovoked seizure relapse in PROSE participants occurred regardless of treatment durations with antiseizure medications. Seven out of 11 participants had their unprovoked seizure despite ongoing treatment, and the remaining four had discontinued their medications in the same temporal range as participants without subsequent unprovoked seizure. Even after weighting cases by their inverse probability of prolonged treatment to compensate for the lack of randomization [28], longer treatments were not associated with reduced risk of unprovoked seizures. Thus, in most cases *with* unprovoked seizure relapse, antiseizure medications in the dose taken were not able to prevent it, whereas in most cases *without* unprovoked seizure, antiseizure treatments turned out unnecessary (Fig. 2). Consequently, in most cases of acute symptomatic seizures, the authors argue for very short durations of antiseizure treatment, e.g., up to 7 days and/or not beyond discharge from the acute care hospital. Exceptions should be made in constellations with high individual risk of further seizures (e.g., persistent CNS infection, recurrent metabolic disturbance, CNS infection causing structural brain damage, acute symptomatic status epilepticus, hypoxic–ischemic encephalopathy), or if even a low-to-moderate risk of seizure relapse cannot be accepted (e.g., high risk of fractures or joint dislocations). In all other cases, watchful waiting seems appropriate, and long-term treatment should not be initiated before a first subsequent unprovoked seizure. As soon as an unprovoked seizure occurs in the presence of a remote brain lesion, the 12-month risk of a further seizure is approximately 40%, and 10-year risk is above 60% [11], so that long-term antiseizure treatment is appropriate.

### Limitations and outlook

Following the definition of acute symptomatic seizures proposed by the ILAE [1], only patients with clinical seizures were recruited for the PROSE register, and no statement can be made on subclinical, electrographic seizures in acute disorders of the brain [42]. Seventeen PROSE participants (12%) had a pathology underlying their acute symptomatic seizure that was not covered by the ILAE definition [1]. However, that definition is not exhaustive [33]. PRES is not included, although acute symptomatic seizures are reported in 50–77% of PRES cases [43, 44], and 11 of the 17 PROSE participants not covered by the ILAE definition had PRES.

Overall, the etiological heterogeneity of the PROSE cohort is both a strength and a limitation of the study, as it allows drawing some rather general conclusions. Disease-specific risk scores of late unprovoked seizure like the ‘SeLECT’ score in ischemic stroke [6, 9] and the ‘CAVE’ score in intracerebral hemorrhage [30] were not taken into account. Neurosurgical conditions like traumatic brain injury, subdural hematoma, and intracranial surgery are underrepresented in the PROSE cohort. To fill this gap, another PROSE cohort restricted to acute symptomatic seizures in neurosurgical conditions is currently being recruited (DRKS00032119). Among non-structural etiologies, drug intoxications and alcohol withdrawals are underrepresented in the PROSE cohort.

All information during the follow-up period was obtained from patients or their caregivers. Therefore, as in all studies on outpatients with epileptic seizures relying on seizure diaries, information on seizure relapse might be inaccurate. Furthermore, office-based physicians’ reasons to continue or discontinue medical treatments were not evaluated.

The PROSE register aimed at studying patients without antiseizure medication or at most with short durations of antiseizure treatment. This was not achieved, although participants might have been treated more deliberately than other patients. Due to the observational nature of the study, the influence of antiseizure medication on seizure relapse risk can be acknowledged but not modified. A randomized interventional trial could compare a very short treatment duration (e.g., 1 week) to a longer treatment (e.g., 3 months). The results of such a trial would provide evidence on whether the use of antiseizure medication beyond discharge from the acute care hospital is helpful at all. If not, the rule of thumb of treating for 3 months can be abandoned, and antiseizure treatments should generally be discontinued during inpatient care to avoid subsequent overtreatment unless certain high-risk features of unprovoked seizure relapse are present.

## Conclusion

This prospective study confirms that even in presence of a structural brain pathology, most acute symptomatic seizures bear a low risk of subsequent unprovoked seizures. Patients received antiseizure medications for longer than recommended in current clinical guidelines, but unprovoked seizures occurred independently from treatment durations. These findings argue for generally restricting the use of antiseizure medications to the acute phase of the underlying disease. However, special caution must be given to individual constellations of increased risk of subsequent unprovoked seizures, e.g., in case of CNS infections with structural affection of brain tissue.

### Supplementary Information

**Additional file 1.** **Table S1:** Seizure screening questions during follow-up interview. **Table S2:** Anonymous online survey on antiseizure treatment strategies. **Table S3:** Patients recruited vs. not recruited for the PROSE register. **Table S4:** Pathologies underlying acute symptomatic seizures with vs. without unprovoked seizure relapse. **Table S5:** Variables associated with prolonged treatment with antiseizure medications, all etiologies. **Table S6:** Variables associated with unprovoked seizure relapse after a first acute symptomatic seizure, all etiologies. **Figure S1:** Modified Rankin score over time. **Figure S2.** Antiseizure treatment strategies of physicians knowing vs. not knowing the PROSE register (.pdf).

**Additional file 2.** Individual responses from the anonymous online survey (.xlsx).

## Data Availability

Upon completion of 3-year follow-up, anonymized participant data of the PROSE register will be made publicly available. Individual answers to the online survey are provided in Additional file 2.

## Abbreviations

CI: Confidence interval
CNS: Central nervous system
DGNI: German neurocritical care society
EEG: Electroencephalogram
IGNITE!: Initiative of German neuro-intensive trial engagement
ILAE: International league against epilepsy
IQR: Interquartile range
MRI: Magnetic resonance imaging
mRS: Modified Rankin scale
PRES: Posterior reversible encephalopathy syndrome
PROSE: Prognosis of acute symptomatic seizures
REDCap: Research electronic data capture
SAPS II: Simplified acute physiology score II
