# Supplementary material for: Prognosis and management of acute symptomatic seizures: a prospective, multicenter, observational study
Source: Ann Intensive Care. 2023 Sep 15;13:85. doi: 10.1186/s13613-023-01183-0 (PMC10504169; doi:10.1186/s13613-023-01183-0)
Supplement: Supplementary file 1 — Additional file 1. Table S1: Seizure screening questions during follow-up interview. Table S2: Anonymous online survey on antiseizure treatment strategies. Table S3: Patients recruited vs. not recruited for the PROSE register. Table S4: Pathologies underlying acute symptomatic seizures with vs. without unprovoked seizure relapse. Table S5: Variables associated with prolonged treatment with antiseizure medications, all etiologies. Table S6: Variables associated with unprovoked seizure relapse after a first acute symptomatic seizure, all etiologies. Figure S1: Modified Rankin score over time. Figure S2. Antiseizure treatment strategies of physicians knowing vs. not knowing the PROSE register (.pdf). [file 13613_2023_1183_MOESM1_ESM.pdf]

Table S1. Seizure screening questions during follow-up interview.

1) In the meantime, have you been diagnosed with epileptic seizures?	
If not:	2a) Have you had other fits / spells, convulsions or falls?
	2b) Have you had uncontrolled movements of part or all of your body such as twitching, jerking, shaking or going limp?
	2c) Have you had episodes with change of awareness or episodes of uncontrolled mental ‘spacing out’?

Questions 2a-2c are modified from Ottman R *et al.* 2010 Epilepsia (doi: 10.1111/j.1528-1167.2009.02274.x). If one of questions 2a-2c was answered ‘yes’, participants were re-interviewed by a neurologist to find out if an epileptic seizure has occurred and whether it was unprovoked. Interviews were conducted in German.

Table S2. Anonymous online survey on antiseizure treatment strategies.

**Welcome**	
Dear colleague,<br>for a research project of the IGNITE! study group of the German Neurocritical Care Society (DGNI), we are conducting a short survey on medical treatment practice of first epileptic seizures. The results shall become part of a scientific publication. You will receive further information at the end of the survey.<br>The survey is addressed to physicians working clinically in Germany. Your participation in the survey is voluntary. The survey is anonymous; therefore, your answers cannot be related to you as a person. IP addresses are not recorded. Please answer to the questions as you would decide in clinical routine. Answering the questions will take approximately 3 minutes of your time. Thank you for your support!	
**Up front, regarding your occupation**	
I work … (multiple answers allowed)	… as a physician in neurology<br>… as a physician in neurosurgery<br>… as a physician in critical care<br>… as a physician in another field<br>- (I do not work as a physician) *
**Fictitious cases. In the following, we will describe four short scenarios.<br>You will be asked as to for how long you recommend antiseizure treatment.<br>Please always choose the answer that best corresponds to your clinical judgement.**	
**Case 1.** In the stroke unit, you are responsible for a 69-year-old, previously healthy female patient with right-sided ischemic stroke (NIHSS 4). Two days after the stroke, she has a single focal to bilateral tonic-clonic seizure originating from the right brain hemisphere. For how long would you give an antiseizure medication, e.g., Levetiracetam?	- Not at all<br>- 1 week<br>- 3 months<br>- 12 months<br>- Permanently
**Case 2.** In the intermediate care unit, you are responsible for a 42-year-old, previously healthy male patient with HSV-1 encephalitis and bi-temporal edema upon neuroimaging. A single bilateral tonic-clonic seizure had led him to admission. For how long would you give an antiseizure medication, e.g., Levetiracetam?	- Not at all<br>- As long as anti-infective treatment is given<br>- 3 months<br>- 12 months<br>- Permanently
**Case 3.** In the rehabilitation unit (post-primary rehabilitation), you are responsible for a 38-year-old, previously healthy male patient with bi-frontal contusion defects 6 weeks after traumatic brain injury. During rehabilitation, he has a bilateral tonic-clonic seizure. For how long would you give an antiseizure medication, e.g., Levetiracetam?	- Not at all<br>- 1 week<br>- 3 months<br>- 12 months<br>- Permanently

**Case 4.** In the intermediate care unit, you are responsible for a 27-year-old, previously healthy first-time mother who has a bilateral tonic-clonic seizure in the context of eclampsia one day after delivery. Her cerebral MRI scan suggests posterior reversible encephalopathy syndrome (PRES). For how long would you give an antiseizure medication, e.g., Levetiracetam?	- Not at all<br>- 1 week<br>- 3 months<br>- 12 months<br>- Permanently
**Acute EEG.** In which of the 4 cases would you have a timely EEG performed and base the duration of antiseizure treatment on its results? (Multiple answers allowed)	- Case 1 (acute stroke)<br>- Case 2 (HSV-1 encephalitis)<br>- Case 3 (rehabilitation after TBI)<br>- Case 4 (eclampsia / PRES)<br>- In none of the cases
**Follow-up EEG.** In which of the 4 cases would you recommend a follow-up EEG and base the duration of antiseizure treatment on its results? (Multiple answers allowed)	- Case 1 (acute stroke)<br>- Case 2 (HSV-1 encephalitis)<br>- Case 3 (rehabilitation after TBI)<br>- Case 4 (eclampsia / PRES)<br>- In none of the cases
**In closing, one question if you already know us.**	
I already know the ‘PROSE register’.	- Yes<br>- No

* Participants answering ‘I do not work as a physician’ were not allowed to continue the survey. The survey was conducted in German.

Table S3. Patients recruited vs. not recruited for the PROSE register.

		**Recruited, n=132 ***	**Not recruited, n=92**	**p, uncorrected**
**Sex**	Female	56 (42%)	37 (40%)	0.78
	Male	76 (58%)	55 (60%)	
**Age [years]**		62 (52-75)	69 (59-80); n=89 **	<u>0.009</u>
**SAPS II**		26 (19-32); n=38 **	29 (23-32); n=32 **	0.14
**Etiology of acute symptomatic seizure**	Structural	114 (86%)	67 (73%)	0.19
	Non-structural	18 (14%)	18 (20%)	
	Not detailed	0	7 (8%)	

Data relate to eligible patients during the multicenter recruitment phase of the study. Data are given as n (column percent) or median (interquartile range). SAPS II, simplified acute physiology score II. * n=133 were recruited; one legal guardian later withdrew consent. ** Otherwise unknown.

Table S4. Pathologies underlying acute symptomatic seizures with vs. without unprovoked seizure relapse.

Pathology	No unprovoked seizure relapse, n=130	Unprovoked seizure relapse, n=11	p, bivariate, uncorrected
Ischemic stroke	35 (27%)	2 (18%)	0.066
Intracerebral hemorrhage	21 (16%)	2 (18%)	
Cerebral venous thrombosis	12 (9%)	2 (18%)	
Subarachnoid hemorrhage	13 (10%)	0	
(P)RES / eclampsia	11 (8%)	0	
Infection + structural affection	10 (8%)	4 (36%)	
Other structural	9 (7%)	1 (9%)	

Data are given as n (column percent) or median (interquartile range). (P)RES, (posterior) reversible encephalopathy syndrome.

Table S5. Variables associated with prolonged treatment with antiseizure medications, all etiologies.

		Medication for <100 days, n=58	Medication for >100 days, n=59	p, bivariate, uncorrected	Odds ratio, multivariable*	p, multivariable*
**Sex**	Female	26 (45%)	27 (46%)	1.0		
	Male	32 (55%)	32 (54%)			
**Age [years]**		65 (56-79)	62 (50-73)	0.15		
**Inpatient treatment**	ICU	26 (45%)	28 (47%)	0.79		
	IMC	8 (14%)	11 (19%)			
	Stroke unit / telemetry unit	18 (31%)	14 (24%)			
	General ward	6 (10%)	6 (10%)			
**Mechanical ventilation**	Ventilation	22 (38%)	15 (25%)	0.17		
	No ventilation	36 (62%)	44 (75%)			
**Sepsis**	Sepsis	4 (7%)	3 (5%)	0.72		
	No sepsis	54 (93%)	56 (95%)			
**Initial mRS**		3 (1-4)	3 (1-5)	0.81		
**Etiology of acute symptomatic seizure**	Structural	46 (79%)	45 (76%)	0.002	1	0.29
	Structural + infectious	1 (2%)	11 (19%)		4.8 (0.6-40.8)	0.16
	Non-structural	11 (19%)	3 (5%)		0.6 (0.1-3.2)	0.56
**Acute symptomatic seizure as initial symptom of underlying pathology**	Initial symptom	30 (52%)	35 (59%)	0.46		
	Not initial symptom	28 (48%)	24 (41%)			

**Delay between manifestation of underlying pathology and seizure**	< 24 hours	48 (83%)	38 (64%)	0.035	1	0.39
	> 24 hours	10 (17%)	21 (36%)		1.6 (0.5-4.6)	
**Acute symptomatic seizure type**	Tonic-clonic	40 (69%)	44 (75%)	0.54		
	Other than tonic-clonic	18 (31%)	15 (25%)			
**Single vs. multiple acute symptomatic seizures**	Single seizure	36 (62%)	42 (71%)	0.36		
	Multiple, within 24 h after first seizure	15 (26%)	9 (15%)			
	Multiple, beyond 24 h after first seizure	7 (12%)	8 (14%)			
**Inpatient medication**	No medication	16 (28%)	4 (7%)	<0.001	1	0.032
	Sedatives only	13 (22%)	0		0	1.0
	Classical antiseizure medication only	11 (19%)	25 (42%)		8.0 (2.0-32.6)	0.003
	Sedatives + classical antiseizure medication	18 (31%)	30 (51%)		5.3 (1.4-20.3)	0.014
**Inpatient EEG**	Epileptiform activity	1 (2%)	8 (14%)	0.002	15.8 (1.4-180)	0.026
	No epileptiform activity	42 (72%)	47 (80%)		5.0 (1.3-19.2)	0.020
	EEG not performed	15 (26%)	4 (7%)		1	0.026

Data are given as n (column percent) or median (interquartile range). mRS, modified Rankin score; EEG, electroencephalogram. * Binary logistic regression; 117 cases included; Nagelkerke's R^2=0.45.

Table S6. Variables associated with unprovoked seizure relapse after a first acute symptomatic seizure, all etiologies.

		No unprovoked seizure relapse, n=130	Unprovoked seizure relapse, n=11	p, bivariate, uncorrected	Odds ratio, multivariable*	p, multivariable*
**Sex**	Female	57 (44%)	2 (18%)	0.12		
	Male	73 (56%)	9 (82%)			
**Age [years]**		62 (52-76)	58 (53-64)	0.29		
**Inpatient treatment**	ICU	63 (48%)	5 (46%)	0.90		
	IMC	19 (15%)	1 (9%)			
	Stroke unit / telemetry unit	35 (27%)	4 (36%)			
	General ward	13 (10%)	1 (9%)			
**Mechanical ventilation**	Ventilation	41 (32%)	4 (36%)	0.74		
	No ventilation	89 (68%)	7 (64%)			
**Sepsis**	Sepsis	8 (6%)	1 (9%)	0.53		
	No sepsis	122 (94%)	10 (91%)			
**Initial mRS**		3 (1-4)	3 (1-5)	0.68		
**Etiology of acute symptomatic seizure**	Structural	101 (78%)	7 (64%)	<u>0.006</u>	1	<u>0.026</u>
	Structural + infectious	10 (8%)	4 (36%)		13.8 (2.0-93.3)	<u>0.007</u>
	Non-structural	19 (14%)	0		0	1
**Acute symptomatic seizure as initial symptom of underlying pathology**	Initial symptom	75 (58%)	3 (27%)	0.060	0.3 (0.1-1.4)	0.13
	Not initial symptom	55 (42%)	8 (73%)		1	

**Delay between manifestation of underlying pathology and seizure**	< 24 hours	98 (75%)	4 (36%)	<u>0.010</u>	1	0.29
	> 24 hours	32 (25%)	7 (64%)		2.2 (0.5-9.3)	
**Acute symptomatic seizure type**	Tonic-clonic	95 (73%)	5 (45%)	0.080	1	<u>0.042</u>
	Other than tonic-clonic	35 (27%)	6 (55%)		5.9 (1.1-32.9)	
**Single vs. multiple acute symptomatic seizures**	Single seizure	80 (62%)	8 (72%)	0.59		
	Multiple, within 24 h after first seizure	29 (22%)	1 (9%)			
	Multiple, beyond 24 h after first seizure	21 (16%)	2 (18%)			
**Inpatient medication**	No medication	20 (15%)	0	0.17		
	Sedatives only	13 (10%)	0			
	Classical antiseizure medication only	37 (29%)	6 (55%)			
	Sedatives + classical antiseizure medication	60 (46%)	5 (45%)			
**Inpatient EEG**	Epileptiform activity	11 (9%)	2 (18%)	0.25		
	No epileptiform activity	99 (76%)	9 (82%)			
	EEG not performed	20 (15%)	0			
**Time to discontinuation of ASM [months]**		3.1 (0.0-9.0); n=109*	3.9 (0.4-9.9); n=4**	0.81		

Data are given as n (column percent) or median (interquartile range). mRS, modified Rankin score; ASM, antiseizure medication. * Binary logistic regression; 141 cases included; Nagelkerke's R^2=0.31. * Otherwise, unknown. ** Otherwise, unprovoked seizure relapse at 3.3 months (0.2-5.4) while still on initial treatment.

Figure S1. Modified Rankin score over time.

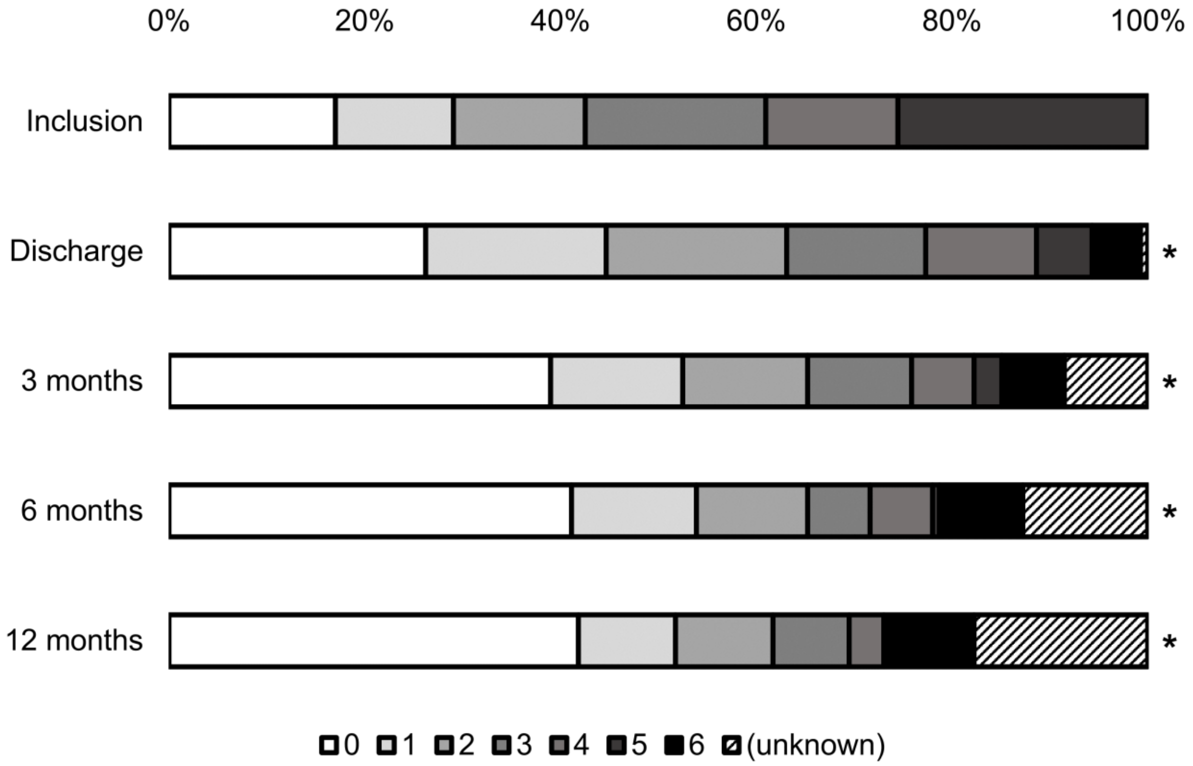

Overall functional outcome according to modified Rankin scale (mRS), obtained at inclusion into the PROSE register (n=141), at discharge from the acute care hospital, and 3, 6, and 12 months after the acute symptomatic seizure. mRS = 0, no symptoms; mRS = 6, death.
* p<0.001 in comparison to time point of inclusion, Wilcoxon test, uncorrected.

Figure S2. Antiseizure treatment strategies of physicians knowing vs. not knowing the PROSE register.

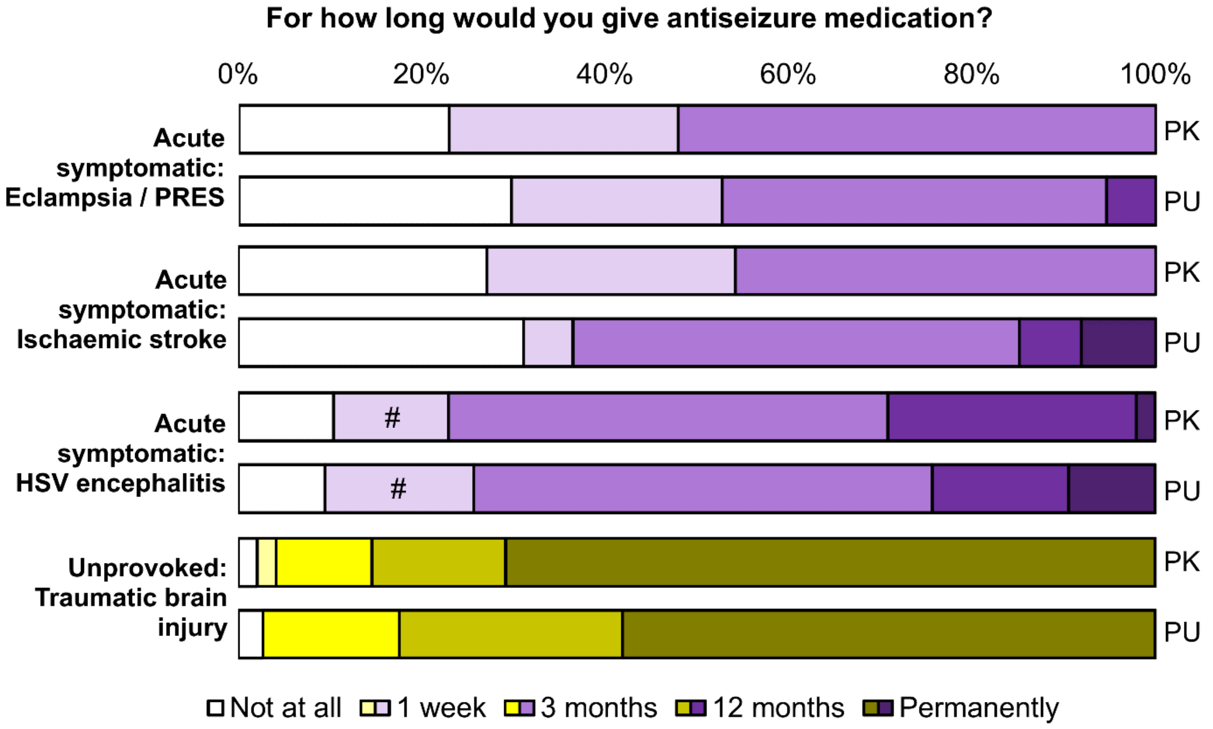

In an anonymous online survey, 122 participating neurologists, neurosurgeons and psychiatrists were asked as to how long they would give antiseizure medication in three fictitious cases of a first acute symptomatic seizure (purple) due eclampsia / posterior reversible encephalopathy syndrome (PRES), ischemic stroke, and herpes simplex virus (HSV) type-1 encephalitis, plus one fictitious case of a first unprovoked seizure (yellow) following traumatic brain injury. Answers of physicians knowing the PROSE register (PK) did not answer significantly different than physicians to whom the PROSE register was unknown (PU; $p>0.05$; Wilcoxon test). [#] In case of HSV encephalitis, the '1 week' option was replaced by 'as long as anti-infective treatment is given'.
